# Effects of the efficacy of the *Yi Shen Tiao Gan* formula on aromatase inhibitor associated musculoskeletal syndrome: a randomized controlled trial

**DOI:** 10.3389/fphar.2025.1639714

**Published:** 2025-07-25

**Authors:** Yan Zhang, Meiling Chu, Meina Ye, Yiqin Cheng, Hui Cong, Yulian Yin, Hongfeng Chen

**Affiliations:** ^1^ Department of Breast Surgery, Longhua Hospital of Shanghai University of Traditional Chinese Medicine, Shanghai, China; ^2^ Department of General Surgery, Seventh People’s Hospital of Shanghai University of Traditional Chinese Medicine, Shanghai, China

**Keywords:** breast cancer, aromatase inhibitor-associated musculoskeletal syndrome, Yishen Tiaogan formula, traditional Chinese Medicine, Western Ontario and McMaster Universities osteoarthritis index, randomized controlled trial

## Abstract

**Background:**

Aromatase inhibitor-induced musculoskeletal syndrome (AIMSS) has emerged as a major cause of treatment discontinuation in hormone receptor-positive patients treated with aromatase inhibitors. There are currently no standardized guidelines or universally accepted treatments for AIMSS. Therefore, this exploratory study aimed to preliminarily evaluate the efficacy and safety of Yi Shen Tiao Gan formula in AIMSS patients.

**Methods:**

A total of 136 patients with AIMSS were included in this single-center, randomized, controlled, single-blind trial and were randomised into the treatment and control groups at a ratio of 1:1. All patients were routinely given Caltrate D. Patients in the treatment group took Breast surgery formula combined with Yi Shen Tiao Gan formula and control group took Breast surgery formula twice a day. The treatment period of Chinese medicine was 3 months as one course of treatment. The clinical efficacy of two courses of treatment was observed in this study. The study observed and compared the following indicators between the two groups: the Western Ontario and McMaster Universities Osteoarthritis Index (WOMAC) scores, Traditional Chinese Medicine symptom and sign scores, bone mineral density, bone metabolism biochemical indicators, bone metabolism-related hormones and safety assessments.

**Results:**

In total, 108 participants were included in the analysis. Before and after treatment, the total WOMAC score of both groups of patients significantly decreased (*p* < 0.05), with the pain subscale score of the treatment group being significantly lower than that of the control group (*p* < 0.05). The TCM symptom and sign score significantly decreased in both groups (*p* < 0.05), with a more pronounced reduction in the treatment group (*p* < 0.05). The bone mineral density T-score of both groups of patients showed a downward trend. The decrease in the left femoral bone density T-score was smaller in the treatment group, while the decline in the lumbar spine bone density T-score was slower in the control group. There was no significant difference in bone metabolism levels between the two groups (*p* > 0.05), but the decrease in β-CTX was slightly slower in the treatment group of the AI + OFS population compared to the control group. After applying Bonferroni correction for multiple testing, these associations did not reach statistical significance. No serious adverse events were observed in either group.

**Conclusion:**

The YSTG formula significantly reduced WOMAC scores, improving pain, stiffness, physical function in patients with AIMSS, and alleviating the traditional Chinese medicine symptoms and signs of liver and kidney deficiency, thereby enhancing the overall quality of life for patients.

**Clinical Trial Registration:**

http://www.chictr.org.cn, identifer ChiCTR2200057785.

## Introduction

According to the International Agency for Research on Cancer, female breast cancer is the second leading cause of cancer incidence globally in 2022, with an estimated 2.3 million new cases, accounting for 11.6% of all cancer cases (Sung et al., 2021). Around 75% of all breast tumors express the estrogen receptor (ER) and/or the progesterone receptor (PR) and are considered as hormone receptor-positive (HR) tumors (Howlader et al., 2014). Adjuvant endocrine therapy remains the mainstay of HR-positive early breast cancer along with chemotherapy and adjuvant radiation therapy. For postmenopausal women, next-generation aromatase inhibitors (AIs) nonsteroidal (anastrozole and letrozole) or steroidal (exemestane) are widely used in the adjuvant setting (Regan et al., 2011). However, administration of AIs has been associated with joint pain and musculoskeletal symptoms that can even lead to treatment discontinuation. Aromatase inhibitor-associated musculoskeletal syndrome (AIMSS) is the most common adverse event encountered by breast cancer patients. In early-stage breast cancer, an incidence of 47% of arthralgias was reported in postmenopausal women treated with AI, including a 23.5% rate of new-onset arthralgia and a 23.5% rate of exacerbation of pre-existing arthralgias (Crew et al., 2007). Another recent meta-analysis showed a 17.9% rate of AI-induced arthralgia in postmenopausal women which was lower in early-stage breast cancer compared with advanced disease (Lammers et al., 2025). Overall, approximately 13%–25% of patients discontinue AI therapy mainly because of arthralgias (Henry et al., 2012). AIMSS has emerged as a major cause of treatment discontinuation in hormone receptor-positive patients treated with AIs. Arthralgias were reported in 13.2%–68.7% of patients receiving AIs for early-stage breast cancer (Skafida et al., 2023). There are currently no standardized guidelines or universally accepted treatments for AIMSS (Grigorian and Baumrucker, 2022). There are no treatments recommended for the management of this iatrogenic disease. Therefore, effective treatment methods for AIMSS are clinically needed.

As an important part of China’s medical and health system, traditional Chinese medicine (TCM) plays an irreplaceable role in diagnosing and treating diseases. TCM has a long history, and after thousands of years of use, it possesses a solid practical foundation and an extensive theoretical system (Sun et al., 2024). Yishen Tiaogan (YSTG) formula is an important TCM treatment for AIMSS. Relevant animal experiments were conducted by this group in the preliminary stage. Our prior research demonstrated that YSTG targets SLIT3 to increase the number of H-type vessel cells in bone, thereby improving Le-induced bone loss flammation and modulating the gut microbiota and we also identified the top 10 chemical components of YSTG formula using ultraperformance liquid chromatography–high resolution mass spectrometry (UPLC–HRMS) (Chu et al., 2025). However, despite these promising findings, there remains a lack of high-quality clinical trials evaluating the efficacy of YSTG formula. Therefore, a comprehensive evaluation of the clinical efficacy of this method is highly important for enriching the theoretical system of TCM treatment.

Therefore, we designed this single-center, randomized, controlled, single-blind trial. In addition to strict inclusion and exclusion criteria, we have also incorporated TCM syndrome scoring to evaluate whether the traditional medical basis of using YSTG formula (TCM syndrome) aligns with improvements in patient musculoskeletal symptoms. Therefore, this exploratory study aimed to preliminarily evaluate the efficacy and safety of YSTG in AIMSS patients.

## Materials and methods

### Study design

This single-center, randomized, controlled, single-blind trial was conducted at the Longhua Hospital affiliated to Shanghai University of Traditional Chinese Medicine from February 2022 to December 2023. Eligible patients were randomly assigned (1:1) to either the YSTG group or the control group. The study protocol was approved by the Ethics Committee of Longhua Hospital affiliated to Shanghai University of Traditional Chinese Medicine (Ethics No. 2022LCSY001), and registered with the Chinese Clinical Trial Registry (ChiCTR2200057785). Prior to commencing treatment in this trial, all participants provided informed consent and agreed to participate in the study, following the guidelines of the Helsinki Declaration. Furthermore, this study strictly adhered to the Consolidated Standards of Reporting Trials (CONSORT) guidelines (Schulz et al., 2010).

### Participants

#### Inclusion criteria

(1) Age ranging from 18 to 65 years old;
(2) Diagnosed with breast cancer by pathology (referring to the 2021 Chinese Society of Clinical Oncology (CSCO) Breast Cancer Diagnosis and Treatment Guidelines (Chinese Anti-Cancer Association Breast Cancer Committee, 2021), all cases were confirmed as breast cancer by basic and molecular pathology), and diagnosed as HR positive by immunohistochemistry;
(3) Completed surgery, chemotherapy, radiotherapy, and/or targeted therapy;
(4) Received AIs (anastrozole, letrozole, or exemestane) for at least 2 months;
(5) Classified as liver and kidney deficiency in TCM syndrome differentiation (referring to the 2002 version of the “Guidelines for Clinical Research of New Chinese Medicines”);
(6) Karnofsky Performance Status (KPS) score ≥60;
(7) Expected survival period exceeding 6 months;
(8) Signed the informed consent form and voluntarily accepted the treatment plan of this study.

#### Exclusion criteria

(1) Bone mineral diseases: such as hypoparathyroidism or hyperparathyroidism, osteitis deformans, osteogenesis imperfecta, osteomalacia, etc.,;
(2) Diseases affecting bone metabolism: such as Cushing’s syndrome, hyperthyroidism, rheumatoid arthritis, etc.,;
(3) Use of steroid hormones or anticonvulsants for more than 6 months before the study;
(4) Hormone replacement therapy within 6 months: such as glucocorticoids, parathyroid hormone, estrogen, etc.,;
(5) Undergone surgery on limb joints within 6 months;
(6) Severe primary diseases of the cardiovascular, liver, kidney and hematopoietic systems;
(7) Obvious disease progression or new distant metastasis during the study;
(8) Patients with cognitive impairment or mental disorders.

#### Shedding/termination criteria

(1) Shedding criteria: Subjects who have completed the informed consent and qualified for screening and entered the randomized trial, but failed to complete the treatment course and observation period as stipulated in the study protocol, will be treated as drop-outs.
(2) Termination criteria: Taking traditional Chinese medicine outside the study; poor medication compliance, not taking traditional Chinese medicine as required; subjects found not meeting the inclusion criteria after enrollment, will be treated as exclusions.

#### Estimation of sample size

This study is a two-group parallel-controlled efficacy clinical trial, with the primary outcome measure being the WOMAC Osteoarthritis Index. The efficacy test for the comparison of means between the two groups is conducted at a significance level of α of 0.05 (two-sided test), with a power (1-β) of 0.8, (calculated according to α = 0.05, β = 0.2, from the table u0.05 = 1.649, u0.2 = 0.817). The recruitment period is 12 months, with a follow-up period of 6 months, and the ratio of the treatment group to the control group is 1:1. The WOMAC questionnaire consists of 24 items, divided into 3 subscales: Pain (5 items); Stiffness (2 items); Physical Function (17 items). The mean and standard deviation of the Pain subscale were (63.13 ± 75.84) for the treatment group and (133.64 ± 95.23) for the control group; for Stiffness, they were (33.00 ± 35.60) for the treatment group and (67.94 ± 45.68) for the control group; for Physical Function, they were (220.21 ± 266.85) for the treatment group and (496.83 ± 338.21) for the control group (Peng et al., 2018). According to the sample size calculation formula, 54 cases are needed per group for the Pain subscale; 48 cases per group for the Stiffness subscale; and 38 cases per group for the Physical Function subscale. Considering all subscales, the required sample size for the WOMAC scale is 54 cases per group, with a dropout rate of 20%, the required sample size is 68 cases per group, ultimately requiring 136 participants. The sample size was estimated according to the formula for the comparison of means between the two groups:

$$\text{nc}=\frac{{\left(Z_{1-\alpha }+Z_{1-\beta }\right)}^{2}\sigma^{2}\left(1+\frac{1}{K}\right)}{{\left(\mu T-\mu C-∆\right)}^{2}}$$

#### Randomization and allocation

SPSS 26.0 statistical software was used to generate random numbers by setting a fixed value of “20,220,101”. The patients were assigned to the treatment group or the control group based on the size of the random numbers at a ratio of 1:1. The enrolled cases were assigned to the corresponding groups in sequence.

Due to the critical nature of AIMSS and the distinct characteristics of traditional Chinese herbal decoctions, blinding of the therapists and patients was not feasible. However, to maintain the integrity of the trial, both the outcome evaluators and data analysts were blinded to the treatment assignments. All data were collected and analyzed by individuals who were unaware of the specific interventions provided to each group.

#### Intervention

The patients were divided into the treatment group and the control group. All patients were routinely given Caltrate D. All patients in the control group were administered the Breast Surgery Formula, which comprises *Salvia chinensis Benth.* (Shi Jian Chuan, 30 g), *Astragalus mongholicus Bunge (*Sheng Huang Qi, 15 g), *Adenophora triphylla (Thunb.) A.DC.* (Nan Sha Shen, 15 g), *Curcuma aromatica Salisb.* (E Zhu, 15 g), *Atractylodes macrocephala Koidz.* (Chao Bai Zhu. 9 g), *Smilax glabra Roxb.* (Fu Ling, 9 g). The treatment group received the YSTG formula in addition to the control regimen. The YSTG formula comprises *Achyranthes bidentata Blume* (Huai niu xi, 30 g), *Eucommia ulmoides Oliv.* (Du zhong, 15 g), *Lycium chinense* (Gou qi zi,15 g), *Cuscuta chinensis Lam.* (Tu si zi, 12 g), *Cyperus rotundus L.* (Zhi xiang fu, 12 g), *Cornus officinalis Siebold and Zucc.* (Shan yu rou, 9 g). All the plant names have been checked with plants of the world online datasets (http://www.plantsoftheworldonline.org). The study medications were provided by the Decoction Room of Longhua Hospital Affiliated to Shanghai University of Traditional Chinese Medicine and were prepared in accordance with the standards set by the Pharmacopoeia of the People’s Republic of China (2015 edition), ensuring compliance with the national drug standards established by the National Medical Products Administration. The components and dosages of Breast Surgery Formula and YSTG formula are listed in Table 1. The quality of the YSTG formula was controlled using ultraperformance liquid chromatography–high resolution mass spectrometry (UPLC–HRMS).

**TABLE 1:** The components and dosage of Breast Surgery Formula and YSTG Formula.

Formula	Chinese names	English names	Latin names	Family	Dosage(g)
Breast Surgery Formula	石见穿 Shi Jian Chuan	Chinese Sage Herb	*Salvia chinensis Benth*	Lamiaceae	30
	生黄芪 Sheng Huang Qi	Mongolian Milkvetch Root	*Astragalus mongholicus Bunge*	Fabaceae	15
	南沙参 Nan Sha Shen	Fourleaf Ladybell Root	*Adenophora triphylla (Thunb.) ADC.*	Campanulaceae	15
	莪术 E Zhu	Blue Turmeric Rhizome	*Curcuma aromatica Salisb*	Zingiberaceae	15
	炒白术 Chao Bai Zhu	Largehead Atractylodes Rhizome	*Atractylodes macrocephala Koidz*	Asteraceae	9
	茯苓 Fu Ling	Indian Buead Tuckahoe	*Smilax glabra Roxb*	Smilacaceae	9
YSTG Formula	怀牛膝 Huai niu xi	Common Achyranthes	*Achyranthes bidentata Blume*	Amaranthaceae	30
	杜仲 Du zhong	Eucommia Bark	*Eucommia ulmoides Oliv*	Eucommiaceae	15
	枸杞子 Gou qi zi	Babury Wolfberry Fruit	*Lycium chinense Mill*	Solanaceae	15
	菟丝子 Tu si zi	Chinese Dodder Seed	*Cuscuta chinensis Lam*	Convolvulaceae	12
	制香附 Zhi xiang fu	Nutgrass Galingale Rhizome	*Cyperus rotundus L*	Cyperaceae	12
	山萸肉 Shan yu rou	Common Macrocarpium Fruit	*Cornus officinalis Siebold and Zucc*	Cornaceae	9

Patients began to take the Chinese herbal decoctions from the first day after enrollment, once a day, divided into two doses, taken 1 hour after meals in the morning and evening. The treatment period of Chinese medicine was 3 months as one course of treatment. The clinical efficacy of two courses of treatment was observed in this study.

Except for the trial medication, no other Chinese medicine was allowed to be used during the observation period. Doctors should require patients to bring all the medications they are currently taking during the visit to check for combined medication. The names (or other therapy names), dosages, frequency and time of any medications that must be continued due to concurrent diseases or other treatments should be recorded in the “Combined Medication Table” of the research medical record for analysis and reporting during the summary.

#### Detection of active components in YSTG formula using UPLC–HRMS

The YSTG formula was identified by Shanghai Lu Ming Biotech Co., Ltd. (Shanghai, China). YSTG formula comprises *Achyranthes bidentata Blume* (Huai niu xi, 30 g), *Eucommia ulmoides Oliv.* (Du zhong, 15 g), *Lycium chinense* (Gou qi zi,15 g), *Cuscuta chinensis Lam.* (Tu si zi, 12 g), *Cyperus rotundus L.* (Zhi xiang fu, 12 g), *Cornus officinalis Siebold and Zucc.* (Shan yu rou, 9 g). They were purchased from the Traditional Chinese Medicine Pharmacy of Longhua Hospital Affiliated to Shanghai University of Traditional Chinese Medicine and identified by Shanghai Lu Ming Biotech Co., Ltd. (Shanghai, China). The quality of YSTG formula was controlled by ultraperformance liquid chromatography–high resolution mass spectrometry (UPLC–HRMS).

A total of 100 mg of a sample was added with 1 mL of water, vortexed for 1 min, sonicated for 60 min, and centrifuged at 4°C for 10 min. All the supernatant was passed through a 0.22 μm water-phase filter membrane and loaded into a sample bottle for analysis. Reference materials were accurately weighed and dissolved in methanol to prepare a mixed standard solution with a concentration of 10 μg/mL. The instrument used was Orbitrap-QE (Thermo Scientific, Karlsruhe, Germany), and the column was ACQUITY UPLC⋅HSS T3 (100 mm × 2.1 mm, 1.8 µm). The mobile phase for solvent A was 0.1% formic acid in water, and solvent B was acetonitrile. The flow rate was set at 0.35 mL/min, the column temperature was maintained at 45°C, and the injection volume was 5 μL.

### Outcomes

#### Primary outcome measure

The primary outcome was the WOMAC (Peng et al., 2018). All patients were assessed 1 day before the traditional Chinese medicine intervention, and then at 3 months ±14 days and 6 months ±21 days after the treatment. The WOMAC consists of 24 items divided into three subscales: Pain (5 items) which includes walking, going up stairs, lying in bed, sitting, and standing; Stiffness (2 items) which includes first thing in the morning and later in the day; and Physical Function (PF) (17 items) which includes the use of stairs, sitting down and getting up, standing, bending, walking, getting in/out of a car, shopping, putting on/off socks, getting in/out of bed, lying in bed, and performing heavy and light housework (Conaghan et al., 2022).

#### Secondary outcome measures

TCM Symptom and Sign Scores: Scores were assigned before and after treatment for symptoms such as soreness and weakness in the waist and knees, lower limb weakness, aversion to cold and cold extremities, leg cramps, increased nocturia, and night sweats, followed by efficacy assessment.

Clinical recovery is defined as the disappearance or near disappearance of clinical symptoms and signs, with a reduction in syndrome scores of ≥95%. Markedly effective: indicates a significant improvement in clinical symptoms and signs, with a decrease in syndrome scores of ≥70%. Effective means an improvement in clinical symptoms and signs, with a reduction in syndrome scores of ≥30%. Invalid refers to no significant improvement or even worsening of clinical symptoms and signs, with a decrease in syndrome scores of less than 30%.

The calculation formula (Nimodipine method) is: $\left[\left(\text{Pre}-\text{treatment score}-\text{Post}-\text{treatment score}\right)÷\text{Pre}-\text{treatment }score\right]\times 100\%.$ $\text{ Efficacy }\text{index }\left(n\right)=\left[\left(preintervention\text{ scores}–preintervention\text{ scores}\right)/\text{preintervention scores}\right]\times 100\%$ .

Bone mineral density (BMD): All subjects underwent BMD testing at the Imaging Department of Longhua Hospital before treatment and 6 months after treatment using Dual X-ray Absorptiometry (DXA), with measurement sites at lumbar spine L1-L4 and the left femur, and the calculation of BMD T-scores.

Bone Metabolism Biochemical Indicators: Total 25-hydroxyvitamin D3 [25(OH)D3], beta C-terminal telopeptide of type I collagen (β-CTX), Osteocalcin (OC), and Growth Hormone (GH).

Bone metabolism-related hormones: Estradiol (Estrogen, E2), Testosterone (Testo).

#### Safety assessments

The interventions will be carried out under the guidance of a researcher. The herbs in the YSTG formula are safe in accordance with the recommended amount in Chinese Pharmacopoeia. We will closely observe the physical conditions and subjective description of the participants. Blood routine, liver and kidney function, estrogen levels, and gynecological ultrasound were tested 1 day before the TCM intervention, and at 3 months ±14 days and 6 months ±21 days after treatment. The occurrence of fractures and gynecological diseases was monitored, and the progression of breast cancer was recorded. Patients who developed distant metastasis were terminated and withdrawn from the study. Unexpected signs or symptoms that occurred during treatment were considered adverse events, and participants were required to report any such events to clinicians during the treatment period.

#### Analytical planning

The intention-to-treat (ITT) analysis was applied to the baseline data, encompassing all participants who were randomized into the study, regardless of their adherence to the treatment protocol. This approach ensures a comprehensive assessment of the initial characteristics of the entire study population. For the primary and secondary outcomes, including the WOMAC scores, bone mineral density, bone metabolism biochemical indicators, bone metabolism-related hormones, and TCM syndrome, statistical analyses were conducted using the per-protocol set (PPS). The PPS included participants who met the inclusion criteria, did not meet the exclusion criteria, and completed the treatment plan as specified. This subset provides a more accurate assessment of the treatment effects under conditions where participants strictly adhered to the study protocol.

#### Statistical analysis

Statistical analysis was performed using IBM SPSS 29.0 software. First, a normality test was conducted on the quantitative data. Data that conformed to a normal distribution were expressed as mean ± standard deviation (±s), and comparisons between groups were made using the independent samples t-test, while pre- and post-treatment comparisons within the same group were made using the paired samples t-test. Data that did not conform to a normal distribution were represented using the median and interquartile range [M (P25, P75)], and the rank sum test was applied. Categorical data were described using percentages, proportions, and ordinal data, and the chi-square test or non-parametric tests were used for analysis. The statistical tests were two-tailed, with a significance level of α = 0.05, and a P-value less than 0.05 was considered to indicate a statistically significant difference. Bonferroni’s *post hoc* test was applied for multiple comparisons to adjust for Type I error inflation due to the simultaneous use of multiple statistical tests.

## Results

### UPLC–HRMS results of YSTG formula

**FIGURE 1:**
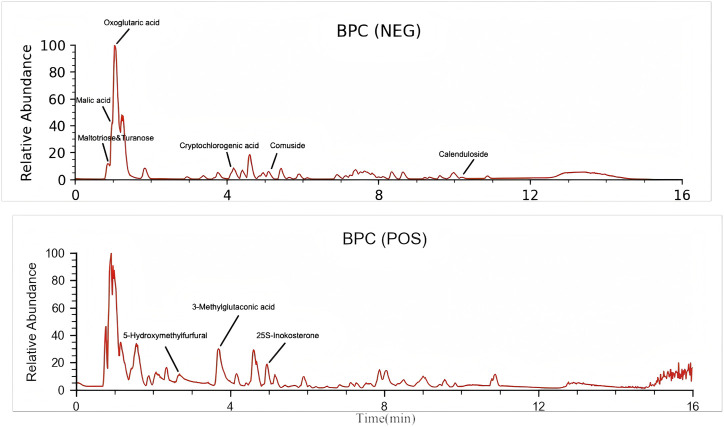
The HPLC analysis of YSTG.

Through UPLC–HRMS analysis in both positive and negative ion modes, a total of 227 compounds were detected. The top 10 chemical components identified in YSTG formula were oxoglutaric acid, 25S-inokosterone, comuside, cryptochlorogenic acid, malic acid, turanose, 5-hydroxymethylfurfural, maltotriose, calenduloside E, and 3-methylglutaconic acid (Figure 1). Detailed information on these components is provided in the Supplementary Material.

### Patient enrollment and baseline characteristics

**FIGURE 2:**
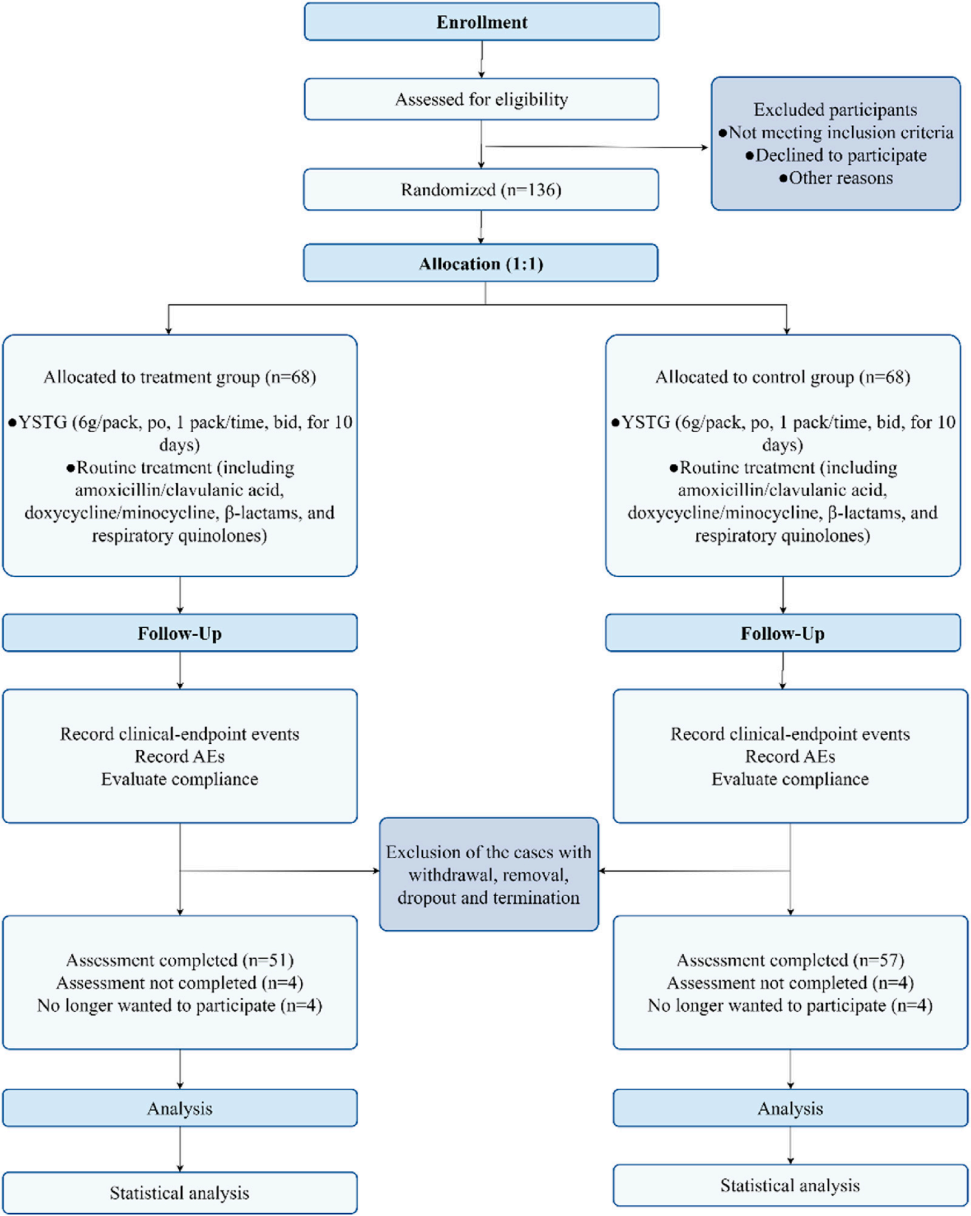
Flow diagram of progress through trial.

From 1 March 2022, to 21 December 2023, a total of 136 AIMSS patients were enrolled in the study and randomly assigned to the treatment group and the control group, with 68 cases in each group. During the study period, 15 patients withdrew, and 13 were excluded. The patients who withdrew were unable or refused to return for follow-up visits due to COVID-19 lockdown policies and personal reasons, and were managed as dropout cases. Among those excluded, seven patients were unable to adhere to the consumption of traditional Chinese medicine or were concurrently using other traditional Chinese medicines; one patient had a low white blood cell count; one patient experienced a recurrence of breast cancer following breast conservation surgery; two patients developed pulmonary and skeletal metastases, respectively; and two patients were excluded after the addition of abemaciclib to avoid interference. Among the 28 patients who were either withdrawn or excluded, 17 were from the treatment group and 11 were from the control group. Finally, 108 participants (treatment group, n = 51; control group, n = 57) completed the trial phase and were analyzed based on PPS (Figure 2). No significant differences were found in the outcome indicators among the two groups (*p* > 0.05) (Tables 2, 3).

**TABLE 2:** Baseline characteristics of the patients (n = 136).

Characteristic		Treatment group (*n* = 68)	Control group (*n* = 68)	*p*
Age (year, mean ± SD)		50.98 ± 7.99	49.66 ± 9.51	0.40
	≤3**0**	1 (1.5%)	1 (1.5%)	
	>30, ≤40	7 (10.3%)	12 (17.6%)	
	>40, ≤50	27 (39.7%)	22 (32.4%)	
	>50, ≤60	25 (36.8%)	24 (35.3%)	
	>60	8 (11.8%)	9 (13.2%)	
Body mass index (kg/m2, mean ± SD)		23.41 ± 3.23	23.09 ± 3.15	0.63
Educational level (N, %)	Junior high school and below	28 (41%)	31 (46%)	0.99
	High school and above	40 (59%)	37 (54%)	
Menarche age (year, mean ± SD)		13.91 ± 1.32	13.72 ± 1.29	0.44
Menopause (N, %)	Pre - menopausal	34 (50%)	37 (54.4%)	0.97
	Menopause duration <10 years	24 (35.3%)	22 (32.4%)	
	Menopause duration ≥10 years	10 (14.7%)	9 (13.2%)	
Gravidity (N, %)	Never pregnant	4 (6%)	6 (9%)	0.99
	Pregnant once	26 (38%)	27 (40%)	
	Pregnant twice	17 (25%)	18 (26%)	
	Pregnant three or more times	21 (31%)	17 (25%)	
Parity (N, %)	Nulliparous	6 (9%)	7 (10%)	0.99
	One delivery	45 (66%)	44 (65%)	
	Two deliveries	17 (25%)	17 (25%)	
Family history of breast cancer (N, %)		13 (19%)	7 (10%)	0.0018
Underlying diseases (N, %)	Hypertension	4 (6%)	6 (9%)	0.99
	Diabetes	2 (3%)	1 (1%)	
	Hyperlipidemia	2 (3%)	1 (1%)	

**TABLE 3:** Baseline oncological characteristics of breast cancer patients (n = 136).

Characteristic		Treatment group (*n* = 68)	Control group (*n* = 68)	*p*
Tumor size (N, %)	≤2 cm	35 (51%)	34 (50%)	0.99
	2.1–5 cm	24 (35%)	26 (38%)	
	>5 cm	9 (13%)	8 (12%)	
Histological Type (N, %)	Non-special type	11 (16%)	9 (13%)	0.99
	Invasive ductal carcinoma	39 (58%)	38 (56%)	
	Invasive lobular carcinoma	5 (7%)	6 (9%)	
	Invasive mucinous carcinoma	5 (7%)	4 (6%)	
	Invasive micropapillary carcinoma	4 (6%)	6 (9%)	
	High-grade ductal carcinoma *in situ*	4 (6%)	5 (7%)	
Histological Grade (N, %)	Grade I	4 (6%)	6 (9%)	0.99
	Grade II	39 (58%)	40 (59%)	
	Grade III	17 (25%)	15 (22%)	
	Grade I–II	3 (4%)	3 (4%)	
	Grade II–III	5 (7%)	4 (6%)	
Molecular Subtype (N, %)	Luminal A type	30 (44%)	24 (35%)	0.89
	Luminal B type (HER2-negative)	26 (38%)	28 (41%)	
	HER2-positive (HR-positive)	7 (10%)	7 (10%)	
Lymph Node Metastasis (N, %)	None	40 (59%)	44 (65%)	0.99
	1–3 nodes	17 (25%)	16 (24%)	
	4–9 nodes	5 (7%)	4 (6%)	
	>10 nodes	6 (9%)	4 (6%)	
Endocrine Therapy (N, %)	Exemestane	38 (56%)	42 (62%)	0.99
	Letrozole	18 (26%)	11 (16%)	
	Anastrozole	12 (18%)	15 (22%)	
Duration of Endocrine Therapy (N, %)	≤6 months	28 (41%)	24 (35%)	0.99
	6–12 months	9 (13%)	13 (19%)	
	13–24 months	21 (31%)	21 (31%)	
	>24 months	10 (15%)	10 (15%)	

### Primary outcome

#### Comparison of WOMAC scores

**FIGURE 3:**
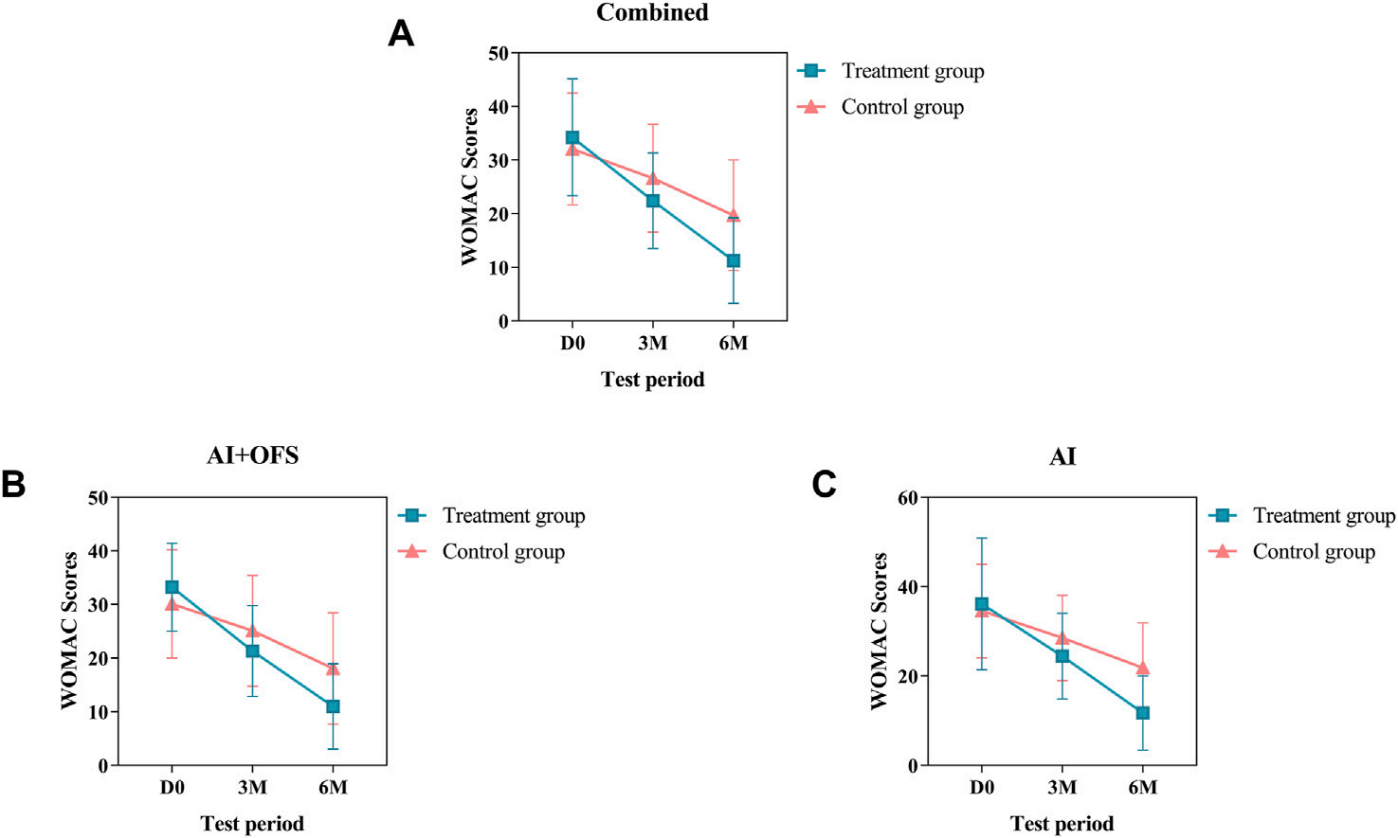
Difference of WOMAC Scores between the treatment and control groups in the change from baseline to 3 and 6 months.

PPS analysis indicated that among 108 patients, at 3 and 6 months post-treatment, the WOMAC total scores were lower in the treatment group compared to the control group [(22.41 ± 8.89 vs. 26.61 ± 10.05, *p* = 0.020, Bonferroni correction) (11.24 ± 7.99 vs. 19.70 ± 10.32, *p* = 0.000, Bonferroni correction)] (Table 4). Further analysis of the subscales (pain, stiffness, physical function) showed that after 6 months of treatment, the scores in these areas were significantly lower in the treatment group than in the control group (*p* < 0.05, Bonferroni correction) (Table 4). Subgroup analysis stratified by endocrine therapy regimen revealed that in both the AI + OFS subgroup and the AI subgroup, the WOMAC total scores and the scores in pain and physical function were significantly lower in the treatment group than in the control group (*p* < 0.05, Bonferroni correction) (Table 4). However, in the AI + OFS subgroup, there was no significant difference in stiffness between the treatment and control groups (*p* > 0.05) (Table 4). Intragroup comparisons between the treatment and control groups and their baselines showed a significant decrease in WOMAC total scores after treatment (*p* < 0.05, Bonferroni correction) (Table 4). Subgroup analysis stratified by endocrine therapy regimen showed that in both subgroups, the WOMAC total scores decreased significantly after treatment (*p* < 0.05, Bonferroni correction), with a more pronounced downward trend observed in the treatment group compared to the control group (Figure 3).

**TABLE 4:** Comparison of WOMAC Scores in the PPS patient population (n = 108) (±s).

Item	Endocrine therapy regimen	3 months		*p*	6 months		*p*
		Treatment group (n = 51)	Control group (n = 57)		Treatment group (n = 51)	Control group (n = 57)	
Pain	AI + OFS	4.21 ± 2.41	5.84 ± 2.30	0.010*	2.48 ± 2.71	4.34 ± 2.36	0.000*
	AI	5.28 ± 2.37	6.20 ± 2.58	0.230	2.44 ± 2.23	4.76 ± 2.86	0.000*
	Combined	4.59 ± 2.43	6.00 ± 2.41	0.000*	2.47 ± 2.52	4.53 ± 2.58	0.000*
Stiffness	AI + OFS	2.61 ± 1.43	2.84 ± 1.39	0.500	1.55 ± 1.12	1.97 ± 1.26	0.160
	AI	2.94 ± 1.35	3.16 ± 0.94	0.560	1.44 ± 1.04	2.32 ± 1.18	0.010*
	Combined	2.73 ± 1.40	2.98 ± 1.22	0.310	1.51 ± 1.08	2.12 ± 1.23	0.010*
PF	AI + OFS	14.48 ± 6.34	16.44 ± 7.52	0.260	6.94 ± 5.14	11.75 ± 7.58	0.000*
	AI	16.22 ± 6.98	19.16 ± 7.06	0.180	7.83 ± 5.98	14.72 ± 7.00	0.000*
	Combined	15.10 ± 6.56	17.63 ± 7.38	0.060	7.25 ± 5.41	13.05 ± 7.42	0.000*
Total	AI + OFS	21.30 ± 8.45	25.12 ± 10.33	0.110	10.97 ± 7.95	18.06 ± 10.36	0.000*
	AI	24.44 ± 9.57	28.52 ± 9.56	0.180	11.72 ± 8.28	21.80 ± 10.10	0.000*
	Combined	22.41 ± 8.89	26.61 ± 10.05	0.020*	11.24 ± 7.99	19.70 ± 10.32	0.000*

*p<0.05.

### Secondary outcome

#### TCM symptom scores and TCM syndrome efficacy

**FIGURE 4:**
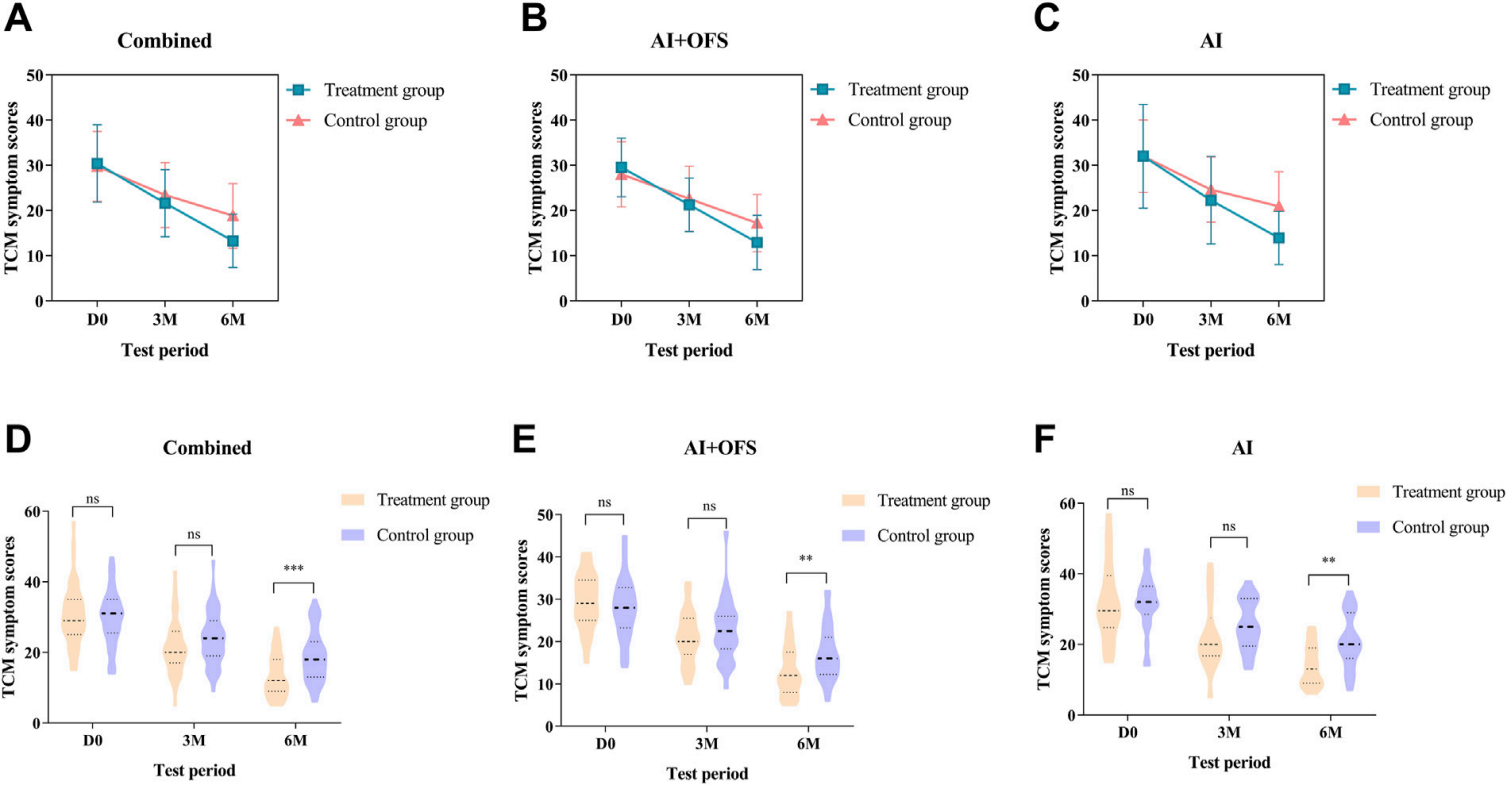
Difference of TCM symptom scores between the treatment and control groups in the change from baseline to 3 and 6 months.

**FIGURE 5:**
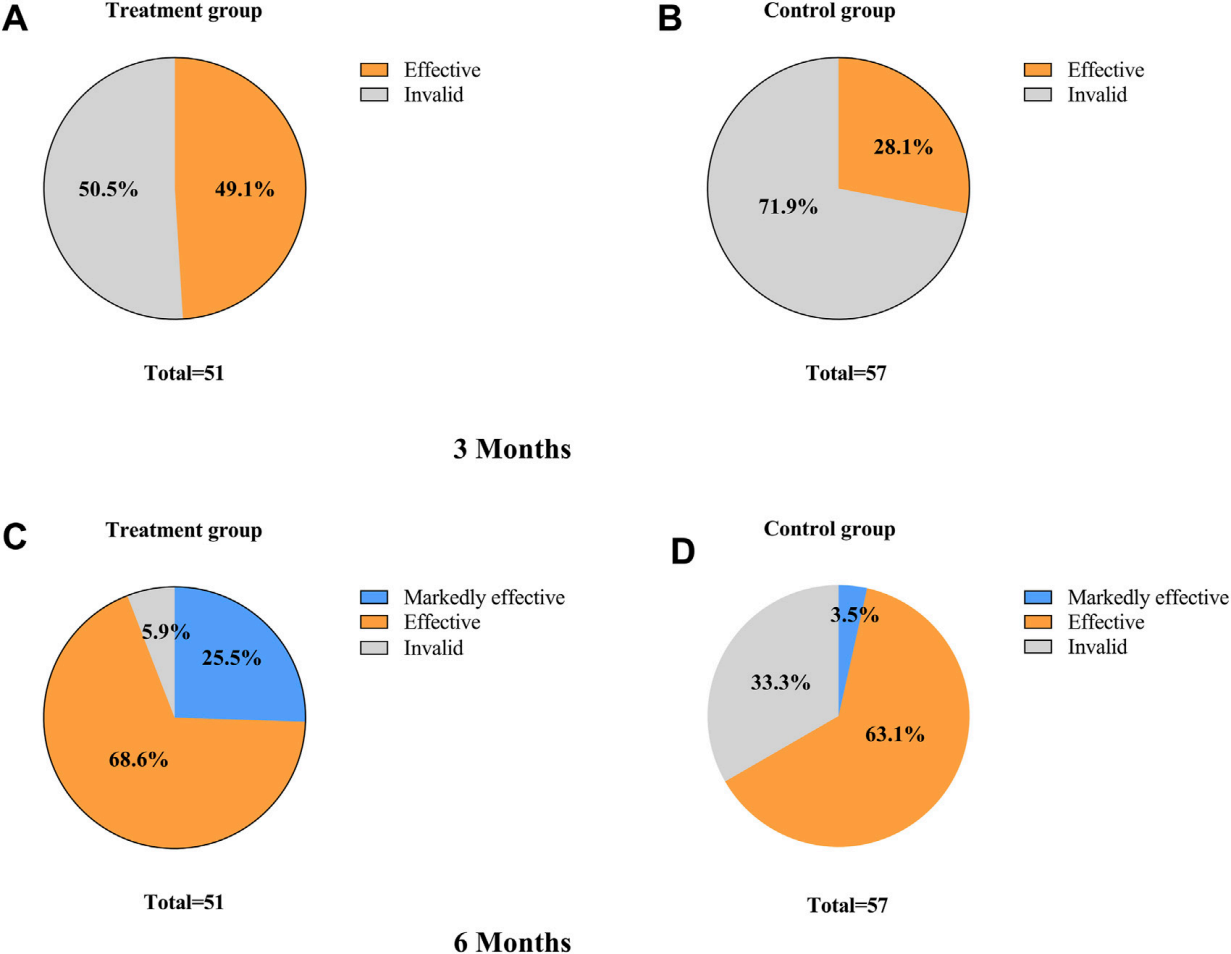
Comparison of Efficacy of TCM symptom efficacy.

After 3 months of TCM treatment, the TCM symptom and sign scores in the treatment group were lower than those in the control group, but the difference was not statistically significant (21.63 ± 7.45 vs. 23.42 ± 7.19, *p* > 0.05) (Table 5). The response rate in the treatment group (49.1%) was significantly higher than that in the control group (28.1%) (*p* = 0.025, Bonferroni correction) (Table 5; Figure 4). Intragroup comparisons showed that scores significantly decreased in both groups, with more pronounced improvement in the treatment group (Figure 4). After 6 months of TCM treatment, the TCM symptom and sign scores in the treatment group were lower than those in the control group (13.29 ± 5.91 vs. 18.84 ± 7.14, *p* = 0.000, Bonferroni correction) (Table 5), and the number of non-responders in the treatment group (3) was significantly less than that in the control group (19) (*p* < 0.001, Bonferroni correction) (Table 6, Figure 5). The markedly effective rate in the treatment group (25.5%) was significantly higher than that in the control group (3.5%) (*p* < 0.001, Bonferroni correction) (Table 6). The scores of both groups significantly decreased compared with the baseline, with a more pronounced improvement trend in the treatment group (Figures 4A–C). Stratified analysis revealed that in the AI + OFS subgroup, the treatment group had better efficacy than the control group (*p* < 0.001, Bonferroni correction) (Figures 4D–F).

**TABLE 5:** Comparison of total score of TCM symptom syndromes in the PPS patient population.

Endocrine therapy regimen	3 months		*p*	6 months		*p*
	Treatment group (n = 51)	Control group (n = 57)		Treatment group (n = 51)	Control group (n = 57)	
AI + OFS	21.26 ± 5.92	22.62 ± 7.19	0.410	12.94 ± 5.99	17.22 ± 6.33	0.010*
AI	22.28 ± 9.69	24.62 ± 7.20	0.400	13.94 ± 5.86	20.92 ± 7.69	0.000*
Combined	21.63 ± 7.45	23.42 ± 7.19	0.220	13.29 ± 5.91	18.84 ± 7.14	0.000*

*p<0.05.

**TABLE 6:** Comparison of TCM Syndrome Efficacy Assessment in the PPS patient population.

Treatment duration	Group	Efficacy assessment				*p*
		Clinical recovery	Markedly effective	Effective	Invalid	
3 months	Treatment group (n = 51)	0	0	25 (49.1)	26 (50.9)	0.025*
	Control group (n = 57)	0	0	16 (28.1)	41 (71.9)	
6 months	Treatment group (n = 51)	0	13 (25.5)	35 (68.6)	3 (5.9)	<0.001*
	Control group (n = 57)	0	2 (3.5)	36 (63.1)	19 (33.3)	

*p<0.05.

### Bone mineral density

**FIGURE 6:**
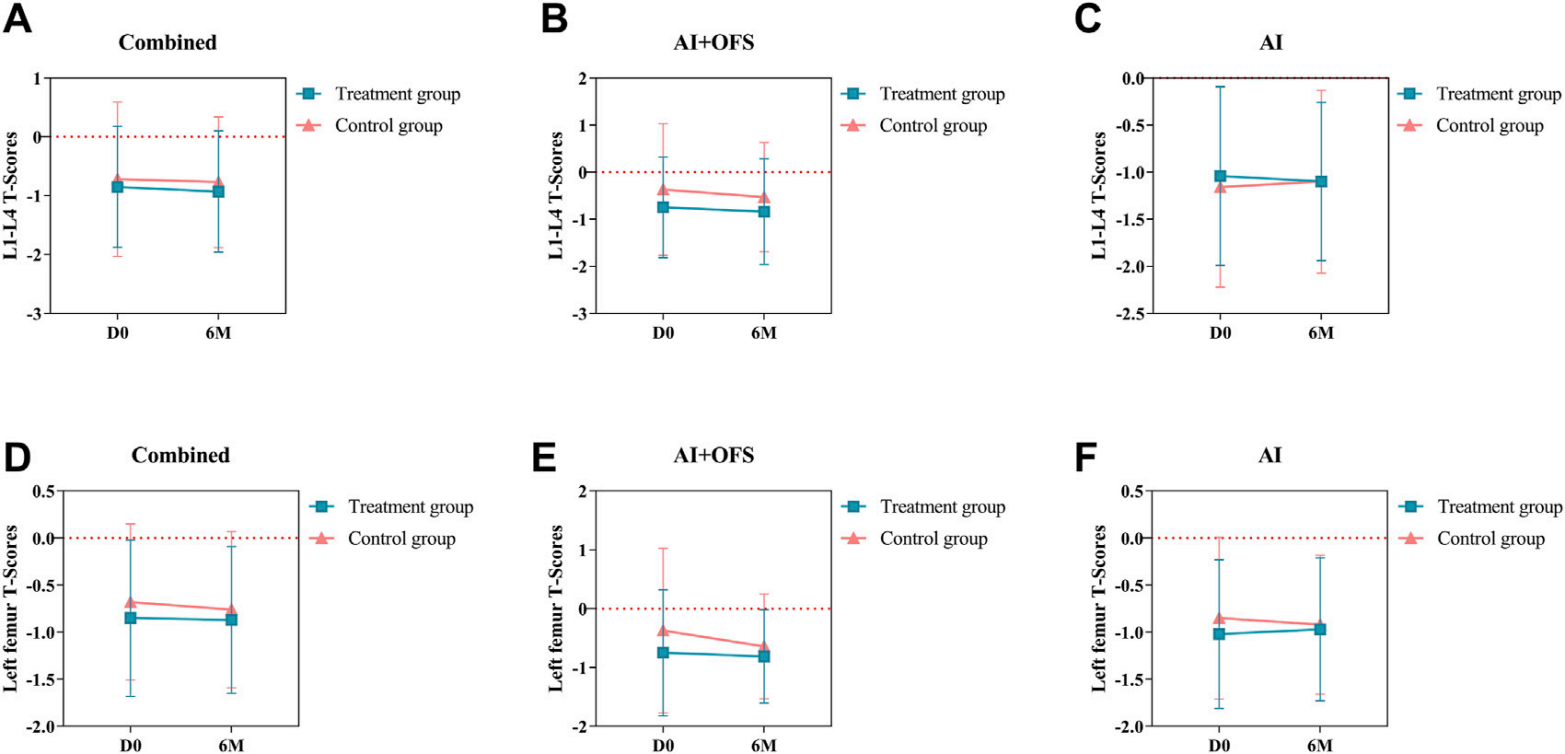
Difference of Bone Mineral Density T-Scores between the treatment and control groups in the change from baseline to 3 and 6 months.

After 6 months of treatment with traditional Chinese medicine, the changes in T-scores of BMD in the lumbar spine and left femur between the two groups were not statistically significant (*p* > 0.05) (Table 7). Intragroup comparisons showed that the BMD T-scores in both groups still tended to decrease, but the differences were not statistically significant (*p* > 0.05) (Figure 6).

**TABLE 7:** Comparison of BMD T-Score**s** in the PPS patient population (n = 108) (±s).

Sites for BMD	Endocrine therapy regimen	Group		*p*
		Treatment group (n = 51)	Control group (n = 57)	
Lumbar Spine (L1-L4)	AI + OFS	−0.84 ± 1.12	−0.53 ± 1.16	0.291
	AI	−1.10 ± 0.84	−1.10 ± 0.97	0.989
	Combined	−0.93 ± 1.03	−0.77 ± 1.11	0.466
Left femur	AI + OFS	−0.81 ± 0.79	−0.64 ± 0.89	0.426
	AI	−0.97 ± 0.76	−0.92 ± 0.74	0.835
	Combined	−0.87 ± 0.78	−0.76 ± 0.83	0.508

### Bone metabolism-related biochemical indicators

**FIGURE 7:**
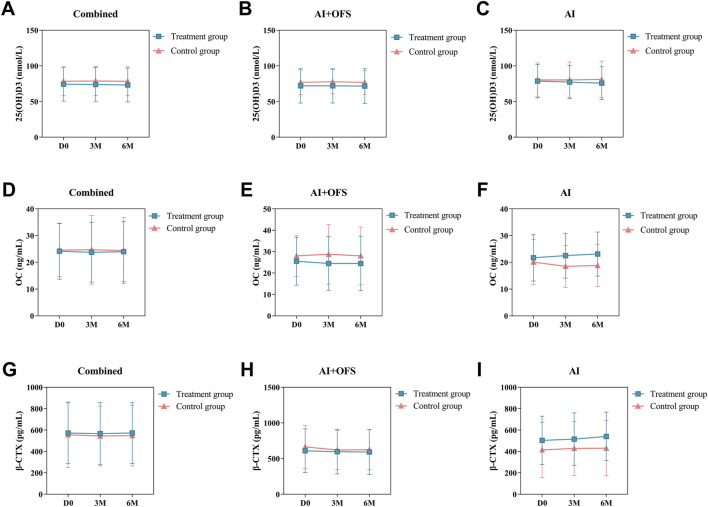
Difference of Bone Metabolism Parameters between the treatment and control groups in the change from baseline to 3 and 6 months.

**FIGURE 8:**
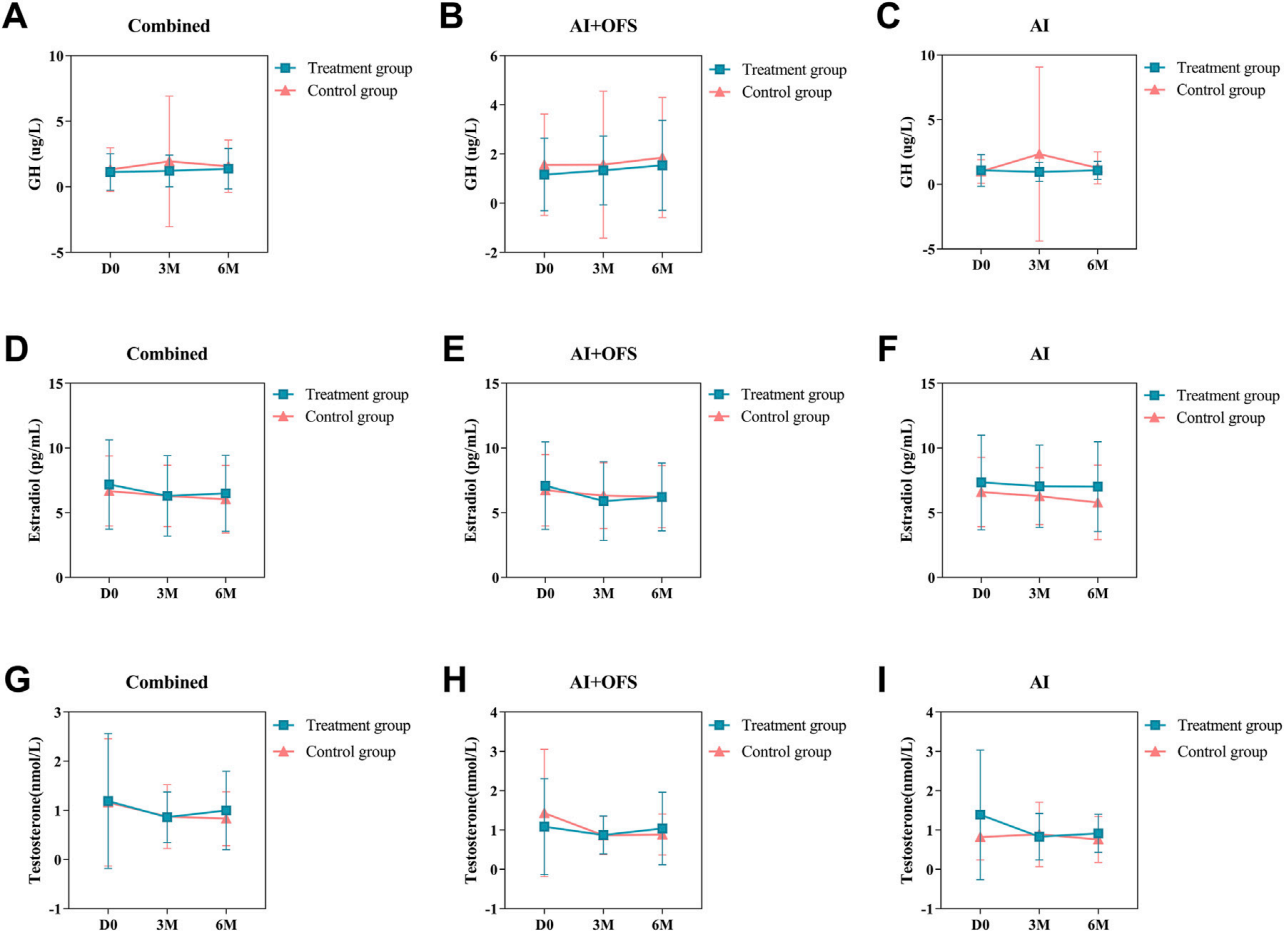
Difference of Bone Metabolism-Related Hormones levels between the treatment and control groups in the change from baseline to 3 and 6 months.

PPS analysis indicated no statistically significant differences (*p* > 0.05) in the levels of bone metabolism biochemical indicators, including 25(OH)D3, β-CTX, OC, GH, Estradiol and Testosterone, between the treatment and control groups at three and 6 months post-treatment (Table 8). Intragroup comparisons showed no statistically significant differences (*p* > 0.05) in bone metabolism levels after treatment in both groups, although β-CTX exhibited a decreasing trend in the treatment group [(567.42 ± 288.65) vs. (544.38 ± 279.96), *p* = 0.68], while an increasing trend was observed in the control group. After 6 months of treatment with TCM, levels of 25(OH)D3 increased in both groups, while OC and β-CTX levels decreased, but these differences were not statistically significant (*p* > 0.05). In the AI + OFS treatment subgroup, the level of growth hormone increased significantly compared to pre-treatment (Figure 7). After 6 months of medication, subgroup analysis stratified by endocrine therapy regimen indicated that the trend of Testosterone level changes in both subgroups shifted from a decreasing trend in the first 3 months to an increasing trend, with the overall decrease in Testosterone levels becoming less pronounced in the treatment group (Figure 8).

**TABLE 8:** Comparison of Bone Metabolism-Related Biochemical Indicators in the PPS patient population (n = 108) (±s).

Item	Endocrine therapy regimen	3 months		*p*	6 months		*p*
		Treatment group (n = 51)	Control group (n = 57)		Treatment group (n = 51)	Control group (n = 57)	
25(OH)D3 (nmol/L)	AI + OFS	72.00 ± 24.04	77.79 ± 17.11	0.280	71.67 ± 24.38	76.78 ± 16.39	0.340
	AI	77.37 ± 23.36	80.37 ± 25.01	0.700	75.79 ± 23.06	81.04 ± 25.47	0.510
	Combined	73.97 ± 23.69	78.81 ± 20.42	0.270	73.16 ± 23.75	78.45 ± 20.30	0.240
OC (ng/mL)	AI + OFS	24.49 ± 12.54	28.77 ± 13.95	0.200	24.47 ± 12.75	28.00 ± 13.46	0.300
	AI	22.51 ± 8.38	18.51 ± 7.82	0.130	23.11 ± 8.23	18.83 ± 7.88	0.120
	Combined	23.76 ± 11.14	24.70 ± 12.85	0.690	23.98 ± 11.25	24.40 ± 12.36	0.860
β-CTX (pg/mL)	AI + OFS	597.42 ± 311.32	620.20 ± 274.91	0.760	591.68 ± 314.97	624.29 ± 278.46	0.670
	AI	515.74 ± 244.43	428.85 ± 251.85	0.280	540.82 ± 226.82	430.27 ± 258.30	0.170
	Combined	567.42 ± 288.65	544.38 ± 279.96	0.680	573.28 ± 284.69	548.21 ± 284.66	0.660
GH (μg/L)	AI + OFS	0.832 (0.4, 1.9)	0.613 (0.2, 1.2)	0.217	0.820 (0.2, 2.2)	1.030 (0.3, 2.0)	0.788
	AI	0.799 (0.4, 1.7)	0.800 (0.2, 1.6)	0.694	1.145 (0.4, 1.7)	0.950 (0.5, 1.3)	0.510
	Combined	0.832 (0.4, 1.7)	0.740 (0.2, 1.5)	0.210	1.010 (0.3, 2.0)	1.030 (0.4, 2.0)	0.723
Estradiol (pg/mL)	AI + OFS	5.000 (5.0, 5.0)	5.000 (5.0, 7.1)	0.379	5.000 (5.0, 6.3)	5.000 (5.0, 6.7)	0.987
	AI	5.000 (5.0, 10.0)	5.000 (5.0, 7.8)	0.523	5.000 (5.0, 10.0)	5.000 (5.0, 5.0)	0.153
	Combined	5.000 (5.0, 5.0)	5.000 (5.0, 7.8)	0.716	5.000 (5.0, 6.4)	5.000 (5.0, 5.0)	0.291
Testosterone (nmol/L)	AI + OFS	0.830 (0.5, 1.1)	0.840 (0.5, 1.2)	0.952	0.970 (0.5, 1.2)	0.770 (0.4, 1.4)	0.657
	AI	0.725 (0.2, 1.4)	0.710 (0.3, 1.1)	0.979	0.800 (0.6, 1.3)	0.550 (0.4, 1.0)	0.137
	Combined	0.830 (0.5, 1.2)	0.790 (0.4, 1.2)	0.765	0.885 (0.6, 1.2)	0.685 (0.4, 1.2)	0.198

### Adverse events

Most subjects well tolerated the research medication, and no serious adverse events were reported. The most common adverse events in both groups were abnormal White Blood Cell (WBC), Alanine Aminotransferase (ALT) and Aspartate Aminotransferase (AST) levels (Table 9). All of the adverse events occurred or disappeared in a short period. No participant discontinued treatment or withdrew due to adverse events. In summary, it is believed that the YSTG formula has no adverse effects on the hematological system, liver and kidney functions, and the prognosis of breast cancer, and the use of the medication is safe.

**TABLE 9:** Summary of adverse events.

Patient ID	Before treatment		3 months		6 months		Explanation
	ALT	AST	ALT	AST	ALT	AST	
052	63	48	51	37	66	49	The patient has a history of elevated liver function over a long period. The hepatology specialist recommended regular follow-ups without medication. During subsequent follow-ups, there was no significant increase in the patient’s liver function indicators
084	46	36	81	45	83	40	After chemotherapy, the patient experienced abnormal liver function indicators. At the time of enrollment, the patient was prescribed Dicycloverine and Eshanfu (a liver protective treatment). During subsequent follow-ups, the patient’s liver function indicators have decreased to within the normal range
118	15	21	33	48	74	149	After chemotherapy, the patient experienced abnormal liver function indicators. At the time of enrollment, the patient was prescribed Dicycloverine and Eshanfu (a liver protective treatment). During subsequent follow-ups, the patient’s liver function indicators have decreased to within the normal range

## Discussion

This single-center, randomized, controlled, single-blind trial evaluated the clinical efficacy and safety of YSTG formula in the treatment of AIMSS and enrolled 136 patients. Notably, this study focuses on the core pathogenesis of “liver stagnation and kidney deficiency” caused by bone and joint side effects of breast cancer endocrine therapy, and it is the first clinical trial in China to explore the efficacy of YSTG formula in treating AIMSS. This study is the first to expand the focus group of aromatase inhibitor-associated musculoskeletal syndrome to include premenopausal patients using AIs in combination with OFS. Our findings demonstrated that the YSTG formula effectively reduced the WOMAC scores and the total points of traditional Chinese medicine symptoms and signs, and significantly improved the clinical symptoms of patients; the treatment had high safety and was well tolerance. As a result, this formula can be used as a complementary treatment for patients with AIMSS.

Endocrine therapy can effectively reduce or inhibit the secretion of estrogen and is the recommended first-line treatment option for HR + breast cancer patients (Tokunaga et al., 2025). Endocrine treatment plans are divided based on the menopausal status of the patient into pre- and post-menopausal categories. AIs are the main drugs for endocrine treatment of post-menopausal HR + breast cancer patients (Fullana et al., 2024). The Early Breast Cancer Trialists’ Collaborative Group (EBCTCG) meta-analysis shows that AIs significantly reduce the 10-year recurrence rate and mortality rate of post-menopausal HR + early-stage breast cancer patients compared to tamoxifen (Early Breast Cancer Trialists’ Collaborative Group EBCTCG, 2015). The efficacy demonstrated by AIs in post-menopausal patients has sparked considerable interest in academia regarding their use in perimenopausal patients. The average age of breast cancer onset in China is relatively low, with about 60% of female patients diagnosed before menopause. Compared to post-menopausal patients, premenopausal women have active ovarian function that continuously secretes estrogen, which may accelerate the proliferation of breast cancer cells (Paluch-Shimon et al., 2022). Ovarian function suppression (OFS) has been widely applied in the treatment of HR + early-stage breast cancer (Pagani et al., 2023). Several long-term follow-up studies have further confirmed the long-term benefits of OFS, showing that it can significantly reduce the distant recurrence risk of premenopausal early-stage breast cancer patients and improve cure rates (Johansson et al., 2022). The SOFT-TEXT study confirmed that for premenopausal patients, AIs combined with OFS significantly improve disease-free survival rates compared to tamoxifen combined with OFS, and continue to reduce the risk of disease recurrence (Pagani et al., 2023). The combination of AIs and OFS is more commonly associated with osteoporosis, fractures, and vaginal dryness, and other adverse events (Hu et al., 2019), which severely affect the quality of life and medication adherence of patients. Currently, there are no clear treatment guidelines domestically or internationally, and clinical interventions often involve bisphosphonates or denosumab, etc. (Skafida et al., 2023). However, these interventions are often costly, have many side effects, and long-term use may lead to drug resistance and increase the incidence of complications. Therefore, it is particularly important to explore the mechanisms of AIMSS in breast cancer patients and new treatment methods.

AI-treated patients mainly report musculoskeletal symptoms such as arthralgias, myalgias, and joint stiffness, which significantly impact their quality of life. A reliable and specific instrument is needed to assess the severity of these symptoms and the efficacy of interventions. The WOMAC score, widely used and well-validated, is highly relevant for our study on improving musculoskeletal symptoms. It directly addresses pain, stiffness, and physical function, making it an ideal primary outcome measure for AIMSS. The WOMAC is a patient-reported outcome (PRO) measure for assessing osteoarthritis of the knees or hips (Zeng et al., 2021). It consists of 24 questions (5 pain, 2 stiffness, and 17 physical function) and can be completed in less than 5 min (Driban et al., 2023). The results indicated that both the treatment group and the control group experienced improvements in musculoskeletal symptoms. The results of the WOMAC score study indicated that both the treatment group and the control group improved in terms of musculoskeletal symptoms. Three months after treatment, the pain subscale score of the treatment group was significantly lower than that of the control group, and the difference between the two groups was more pronounced among those receiving AI plus OFS therapy. Intragroup comparisons revealed that the control group showed a significant decrease in overall WOMAC scores; the treatment group demonstrated a clear downward trend in WOMAC scores both overall and after stratified analysis. Six months after treatment, when comparing WOMAC scores between the two groups and conducting stratified analysis, the results showed that at all levels of comparison, the treatment group had lower scores than the control group. Intragroup comparisons indicated that both groups experienced a significant decrease in WOMAC scores after overall analysis and stratified statistics.

AIs cause bone loss and osteoporosis, which are primarily manifested clinically as joint pain, bone pain, morning stiffness, and other symptoms. These can be categorized under the TCM concept of “bone impediment”, and their pathogenesis is closely related to the kidney, liver, and blood stasis. Previous studies have found that using kidney-tonifying methods can increase patients’ lumbar spine and femoral bone density, and can also raise the levels of PINP and lower the levels of β-CTX (Yin et al., 2018). However, the side effects of AIs are not limited to the reduction of bone mass; they also include bone and joint pain; moreover, the age range of those using AIs is broad, and their physical conditions vary. If kidney-tonifying therapy is used as a universal treatment, it cannot accommodate all syndrome types and does not reflect the TCM principle of “treating the same disease differently based on varying conditions.” This study, starting from TCM theory, treats AIMSS based on the theory of liver depression and kidney deficiency, broadening the clinical treatment approach for AIMSS. The main components of the Yishen Tiao Gan formula include Achyranthes bidentata (Huai niu xi), Eucommia ulmoides (Du zhong), Lycium barbarum (Gou qi zi), Cuscuta chinensis (Tu si zi), Cyperi Rhizoma (Zhi xiang fu), and Cornus officinalis (Shan yu rou). Previous studies have found that the YSTG formula may improve bone loss by up-regulating SLIT3 to induce H-type angiogenesis. Additionally, modern medical research has demonstrated that Achyranthes bidentata polysaccharide suppresses osteoclastogenesis and bone resorption via inhibiting RANKL signaling (Song et al., 2018). Eucommia ulmoides Oliver polysaccharide alleviates glucocorticoid-induced osteoporosis by stimulating bone formation via ERK/BMP-2/SMAD signaling (Song et al., 2024). LBP1C-2 from Lycium barbarum alleviated age-related bone loss by targeting BMPRIA/BMPRII/Noggin (Sun et al., 2023). The active components in Cuscuta chinensis Lam effectively prevent and treat postmenopausal osteoporosis by modulating bone marrow macrophage polarization through the NF-κB/IκBα signaling pathway (Li et al., 2025).

Significant improvements in symptoms were observed in both groups in terms of the TCM symptom and sign score. After 3 months of medication, the treatment group had an effectiveness rate of 49.1%, and both groups showed a marked decrease in TCM symptom and sign scores, with a more pronounced downward trend in the treatment group. As the treatment time increased, the effect became more apparent. After 6 months of treatment, the effectiveness rate in the treatment group reached 68.6%, and the TCM symptom and sign scores in the treatment group were significantly lower than those in the control group, with a more noticeable downward trend in the treatment group. This has clinical guidance significance for the treatment of AIMSS patients with TCM.

The management of AIMSS in breast cancer patients needs a multidisciplinary approach integrating pharmacological and non-pharmacological supportive care strategies (Piazzon et al., 2025). Endocrine therapy with AIs is key for HR + breast cancer but causes significant musculoskeletal side effects. Current pharmacological interventions for AIMSS often involve bisphosphonates or denosumab, which can be costly and have side effects. The YSTG formula offers a potential alternative with high safety and efficacy. Non-pharmacological interventions like physical therapy, exercise programs, and psychological support also play a crucial role in managing AIMSS (Piazzon et al., 2025). Integrating the YSTG formula with these non-pharmacological strategies can enhance overall patient outcomes.

The study still has some limitations as follows. First, the study was not designed as a double-blind trial. The traditional double-blind double-mimic method is not affordable and suitable for this study of multiple TCM prescriptions. Therefore, we finally chose single-blind which conforms to the complexity and feasibility of TCM and implemented it strictly to control the risk of reveals. Secondly, our study did not include a placebo control for YSTG formula due to ethical considerations and the need to provide active treatment to all participants. The Breast Surgery Formula, used in both groups, was designed to address postoperative symptoms and complications in breast cancer patients. This approach, while ensuring ethical standards, limits our ability to definitively attribute observed effects solely to the YSTG formula. Future studies should consider a placebo control and separate groups receiving either the Breast Surgery Formula alone, the YSTG formula alone, or the combined treatment to better assess the independent and combined effects of each formula. Additionally, the three- and 6-month treatment durations may be inadequate for capturing the complete bone metabolism cycle, and this short duration could mask differences in BMD and other relevant indicators. Therefore, future studies should extend the treatment and follow-up periods to better evaluate the long-term efficacy of YSTG formula. Second, the small sample size and the selection of patients exclusively from one hospital may have introduced bias. Therefore, future studies with larger sample sizes from multiple centres are needed to address the present limitations.

## Conclusion

The YSTG formula significantly reduced WOMAC scores, improving pain, stiffness, physical function in patients with AIMSS, and alleviating the traditional Chinese medicine symptoms and signs of liver and kidney deficiency, thereby enhancing the overall quality of life for patients. Despite the limitations of this study, it provides evidence-based medicine for the treatment of AIMSS with Chinese herbs, potentially offering a new alternative to treatments such as bisphosphonates or denosumab. Future research should focus on the broader integration of supportive care strategies for AIMSS. This includes exploring the synergistic effects of combining the YSTG formula with other pharmacological and non-pharmacological interventions. Such research will contribute to the development of comprehensive, evidence-based guidelines for the management of AIMSS in breast cancer patients.

## Data Availability

The original contributions presented in the study are included in the article/Supplementary Material, further inquiries can be directed to the corresponding authors.
